# Supplementary material for: Replication of Equine arteritis virus is efficiently suppressed by purine and pyrimidine biosynthesis inhibitors
Source: Sci Rep. 2020 Jun 22;10:10100. doi: 10.1038/s41598-020-66944-4 (PMC7308276; doi:10.1038/s41598-020-66944-4)
Supplement: Supplementary file 1 — Supplementary information. [file 41598_2020_66944_MOESM1_ESM.docx]

**Replication of Equine arteritis virus is efficiently suppressed by purine and pyrimidine biosynthesis inhibitors.**

José Carlos VALLE-CASUSO^1*^, Delphine GAUDAIRE^1*^, Lydie MARTIN-FAIVRE^1^, Anthony MADELINE^1^, Patrick DALLEMAGNE^2^, Stéphane PRONOST^3^, Hélène Munier-Lehmann^4^, Stephan ZIENTARA^5^, Pierre-Olivier VIDALAIN^6,7,^ and Aymeric HANS^1^.

^1^ Laboratoire de Santé Animale, site de Normandie de l’ANSES, PhEED Unit, 14430 Goustranville, France.

^2^ Normandie Univ, UNICAEN, CERMN EA4258, 14000 Caen, France

^3^ LABÉO Frank Duncombe, Normandie Univ, UNICAEN, BIOTARGEN EA7450, 14280 Saint-Contest, France.

^4^ Institut Pasteur, Unité de Chimie et Biocatalyse, CNRS UMR 3523, 75015 Paris, France

^5^ Université Paris-Est, Laboratoire de Santé Animale, ANSES, INRA, ENVA, UMR 1161 Virologie, 94700 Maisons-Alfort, France

^6^ Equipe Chimie et Biologie, Modélisation et Immunologie pour la Thérapie (CBMIT), Université Paris Descartes, CNRS UMR 8601, 75006 Paris, France.

^7^ Centre International de Recherche en Infectiologie, INSERM U1111, CNRS UMR5308, Université Lyon 1, ENS de Lyon, Lyon, France

* These authors contributed equally to this work.

Corresponding author

Aymeric HANS ([aymeric.hans@anses.fr](mailto:aymeric.hans@anses.fr))


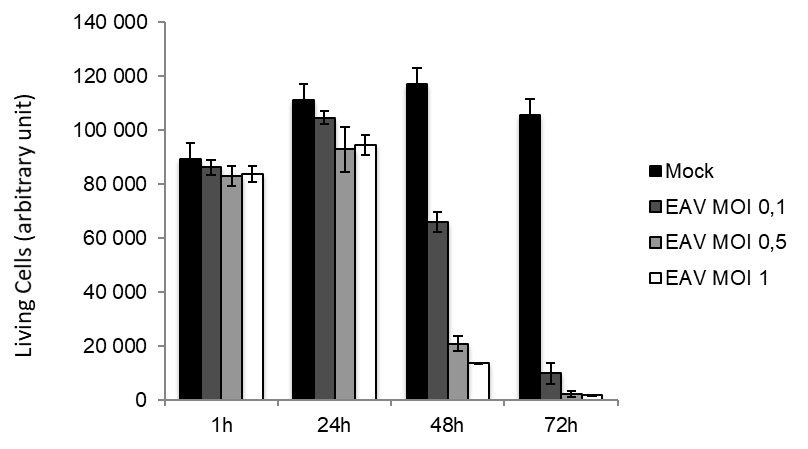


Supplementary Figure S1
